# *Dermacentor reticulatus*, a putative vector of *Babesia* cf. *microti* (syn. *Theileria annae*) piroplasm

**DOI:** 10.1007/s00436-017-5379-0

**Published:** 2017-01-23

**Authors:** Adnan Hodžić, Johanna Zörer, Georg Gerhard Duscher

**Affiliations:** Institute of Parasitology, Department of Pathobiology, University of Veterinary Medicine Vienna, Veterinaerplatz 1, 1210 Vienna, Austria

**Keywords:** *Babesia* cf. *microti*, *Theileria annae*, *Dermacentor reticulatus*, Potential vector, PCR, Sequencing

## Abstract

*Babesia* cf. *microti* (syn. *Theileria annae*, *Babesia microti*-like, *Babesia vulpes*) is a recently recognized tick-borne piroplasm that infects domestic and wild carnivores. Although *Ixodes hexagonus* is considered as the leading candidate responsible for the transmission, its capacity to act as a competent vector has not yet been confirmed. This study reports the occurrence of *B.*cf. *microti* in unfed *Dermacentor reticulatus* for the first time, suggesting that this tick species may be implicated in the life cycle of this canine parasite. Out of 128 questing *D. reticulatus* ticks collected in eastern Austria, nine (7%) and four (3%) of them were found to be PCR positive for *B. canis* and *B.* cf. *microti*, respectively. Although the data presented here are not sufficient to explicitly state that *D. reticulatus* is a competent vector of *B.* cf. *microti*, our results can at least give a hint for future studies, which need to include experimental transmission in order to confirm its vector competence and possible involvement in the transmission of this babesial species.

## Introduction

*Babesia* cf. *microti* is a protozoan tick-transmitted parasite affecting domestic dogs and wild carnivores worldwide (Alvarado-Rybak et al. 2016). It is also known as *Theileria* (*Babesia*) *annae*, *Babesia* sp. “Spanish dog isolate” and *Babesia vulpes*, but due to lack of an appropriate description, all these names are considered unavailable (Harris 2016). Thus, given that this canine piroplasm is genetically most closely related to zoonotic *B. microti* and that current valid name is not yet available, it can only be informally referred as “microti group” (Harris 2016). Infection by *B.* cf. *microti* in dogs can cause severe disease with clinical signs of hemolytic regenerative anemia, thrombocytopenia, azotemia, anorexia, lethargy, renal failure, or even death (Zahler et al. 2000; Falkenö et al. 2013; Miró et al. 2015), while clinical impact of the parasite in wild carnivores still remains abstruse (Clancey et al. 2010; Miró et al. 2015).

The vector of *B.* cf. *microti* is currently unknown, but hedgehog tick *Ixodes hexagonus* has been proposed as the main candidate responsible for its transmission, based solely on the associations between the occurrence of this tick species and infection in dogs (Camacho et al. 2003). However, the detection of *B.* cf. *microti* in domestic and wild canids originating from areas lacking *I. hexagonus* (Birkenheuer et al. 2010; Falkenö et al. 2013) may indicate the possible involvement of other ixodid ticks in the transmission cycle of this protozoon. Nevertheless, the piroplasmid DNA has been detected in engorged *I. ricinus*, *I. canisuga*, *I. hexagonus*, and *Rhipicephalus sanguineus* ticks collected from different animals (Iori et al. 2010; Lledó et al. 2010; Najm et al. 2014). However, their competence to serve as vectors of *B.* cf. *microti* has not been proven, and the DNA present in the ticks may be a result of the current blood meal (Najm et al. 2014). As in case of other canine babesial species, i.e., *B. canis* and *B. gibsoni*, the non-vectorial routes of transmission including transplacental transmission and direct infection by dog bite can also occur in *B.* cf. *microti* (Yeagley et al. 2009; Simões et al. 2011).

In this study, we report the detection of *B.* cf. *microti* piroplasm in unfed adults of *D. reticulatus* collected in eastern Austria and also discuss its potential role as vector of this little known parasite.

## Material and methods

**Fig. 1:**
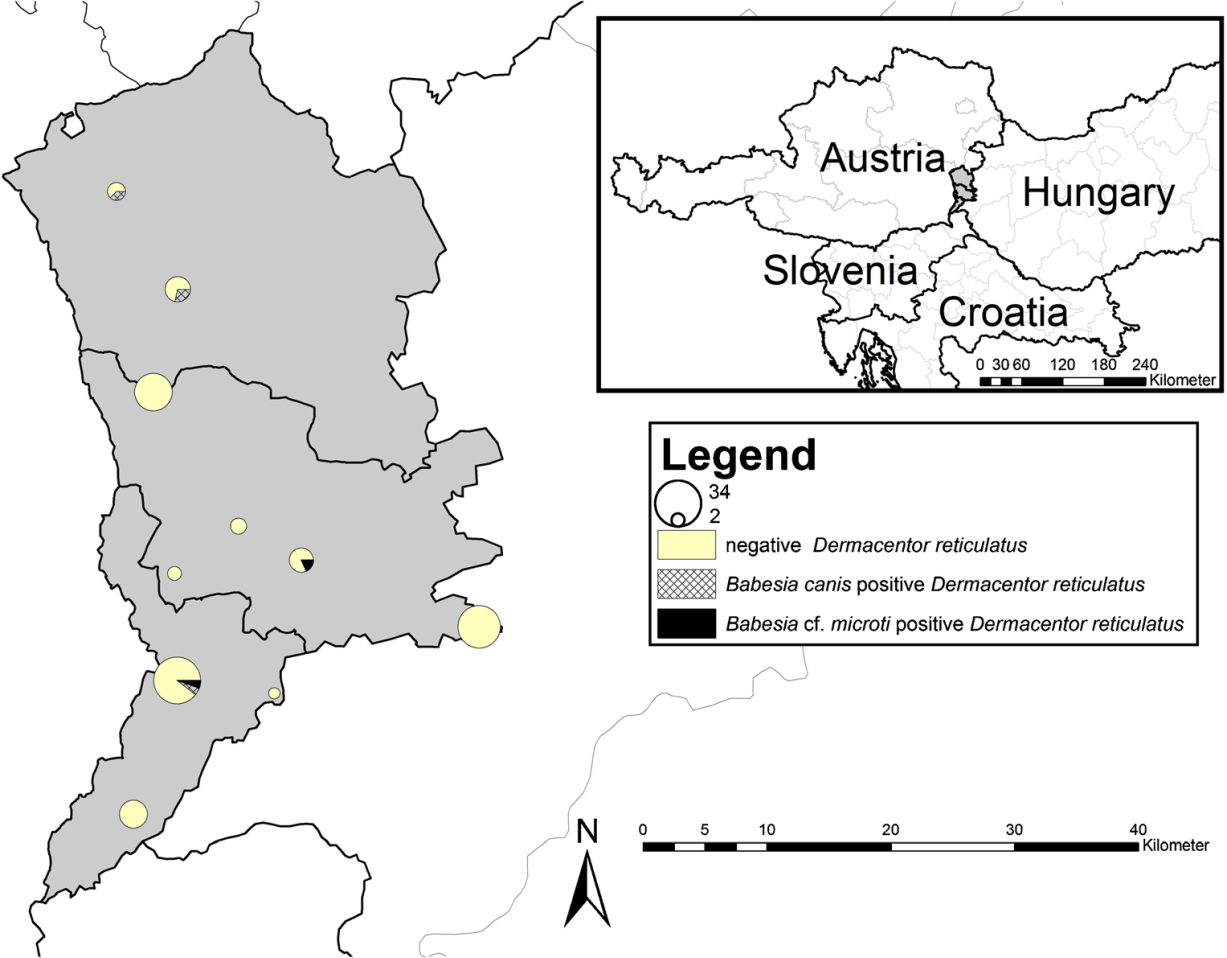
Sampling area and sites with negative, *Babesia canis* and *Babesia* cf. *microti* positive *Dermacentor reticulatus*. The size of the *circles* is proportional to the number of *ticks* sampled

In March, April, and September 2016, questing *D. reticulatus* ticks were collected from several suitable locations in Burgenland, eastern Austria by flagging vegetation (Fig. 1). After the morphological identification, all specimens were processed individually for DNA extraction following the procedure previously described (Hodžić et al. 2016). The quality of tick DNA was assessed by PCR amplification of the tick mitochondrial 16S ribosomal ribonucleic acid (rRNA) gene (Halos et al. 2004). For molecular detection and characterization of *Babesia* and *Theileria* species, two sets of primers were used to amplify 561-bp-long fragment of 18S rRNA gene in nested PCR reaction (Zintl et al. 2011). Amplified PCR products were visualized by agarose gel electrophoresis, purified, and sequenced in both directions (Mycrosinth, Austria). The sequences were aligned and compared for similarity with those available in GenBank® database using Basic Local Alignment Search Tool (BLAST) analysis.

## Results and discussion

Out of total 128 adult specimens of *D. reticulatus* collected, 13 (10%) were found to be positive with *Babesia*/*Theileria* specific nested PCR. Sequence analysis and BLAST search of nine samples (7%) revealed 100% identity to the sequences of *B. canis* found in unfed adult of *D. reticulatus* (GenBank® accession no. AY649326) and domestic dogs (e.g., KT008057). The remaining four sequences (3%) were 100% identical to *B.* cf. *microti* previously identified in foxes from Austria (KM115972, KM115968), Bosnia and Herzegovina (KP216411), Great Britain (KT580785), and foxes (KT223483) and dogs (e.g., EU583387, AY457974) from endemic regions in Spain. The nucleotide sequences herein obtained have been deposited in GenBank® database and are available under accession numbers KY447296 (*B. canis*) and KY447297 (*B.* cf. *microti*).

To the best of our knowledge, this is the first report of *B.* cf. *microti* piroplasm in unfed adults of *D. reticulatus*. Although the ticks could acquire the parasite at the nymphal stage by feeding on an infected animal host as already implied for *Hepatozoon canis* (Giannelli et al. 2013), it is not unreasonable to propose this tick species as a potential vector of *B.* cf. *microti*. First of all, *D. reticulatus* is considered as an ideal arthropod vector due to its extraordinary biological features, such as high reproduction rate, rapid development cycle, ability to survive in different environments, and capacity to host and transmit a wide range of pathogens of medical and veterinary concern, including *Babesia* and *Theileria* species of canids and equids (Földvári et al. 2016). Furthermore, it has recently been suggested as vector of closely related *B. microti* in Europe (Wójcik-Fatla et al. 2012). In addition, *D. reticulatus* is one of the most reported tick species in Europe (Földvári et al. 2016), and among other, it feeds on domestic and wild carnivores (e.g., dogs, foxes, wolves) that serve as hosts for *B.* cf. *microti* pathogen. Also, it was the most common tick after *I. hexagonus* found in dogs infected by *B.* cf. *microti* in hyperendemic region in northwestern Spain (Camacho et al. 2003). Last but not least, the fact that the geographical distribution of *D. reticulatus* in Europe closely overlaps with the distribution of *B.* cf. *microti* (see Alvarado-Rybak et al. 2016; Földvári et al. 2016), as well as that the same sequence type identified in host-seeking *D. reticulatus* ticks is also circulating among vertebrate hosts, supports the existence of the possible tick-host-pathogen association. Recently, we identified 50% of the foxes to harbor this pathogen in a close by area little further north (Duscher et al. 2014), emphasizing the occurrence in wildlife consequently bearing a potential impact on domestic dogs.

In the present study, *B.* cf. *microti* DNA was detected in unfed *D. reticulatus* ticks, suggesting that this tick species might be a competent vector for this canine piroplasm. Although the data presented here are not sufficient to explicitly support this hypothesis, our results can at least give a hint for future studies which need to include experimental transmission in order to confirm its vector competence and implication in the transmission of *B.* cf. *microti*.
